# Gait disturbances as specific predictive markers of the first fall onset in elderly people: a two-year prospective observational study

**DOI:** 10.3389/fnagi.2014.00022

**Published:** 2014-02-25

**Authors:** Jean-Baptiste Mignardot, Thibault Deschamps, Eric Barrey, Bernard Auvinet, Gilles Berrut, Christophe Cornu, Thierry Constans, Laure de Decker

**Affiliations:** ^1^Laboratory “Motricité, Interactions, Performance” (UPRES EA 4334), University of NantesNantes, France; ^2^Up-COURTINE Lab, Centre for Neuroprosthetics and Brain Mind Institute, Ecole Polytechnique Fédérale de LausanneLausanne, Switzerland; ^3^Unité de Biologie Intégrative des Adaptations à l'Exercice (Inserm U902) Genople, Université d'Evry Val d'EssonneÉvry, France; ^4^GABI, UMR-1313, INRAJouy-en-Josas, France; ^5^Service de Rhumatologie, Centre Hospitalier de LavalLaval, France; ^6^Gérontopôle des Pays de la Loire, CHU de NantesNantes, France; ^7^Geriatrics Department, Centre Hospitalier Universitaire de ToursTours, France

**Keywords:** risk of fall, gait analysis, gait variability, gait speed, accelerometric device, fall-related injuries, home-dwelling people, principal components analysis

## Abstract

Falls are common in the elderly, and potentially result in injury and disability. Thus, preventing falls as soon as possible in older adults is a public health priority, yet there is no specific marker that is predictive of the *first* fall onset. We hypothesized that gait features should be the most relevant variables for predicting the first fall. Clinical baseline characteristics (e.g., gender, cognitive function) were assessed in 259 home-dwelling people aged 66 to 75 that had never fallen. Likewise, global kinetic behavior of gait was recorded from 22 variables in 1036 walking tests with an accelerometric gait analysis system. Afterward, monthly telephone monitoring reported the date of the first fall over 24 months. A principal components analysis was used to assess the relationship between gait variables and fall status in four groups: non-fallers, fallers from 0 to 6 months, fallers from 6 to 12 months and fallers from 12 to 24 months. The association of significant principal components (PC) with an increased risk of first fall was then evaluated using the area under the Receiver Operator Characteristic Curve (ROC). No effect of clinical confounding variables was shown as a function of groups. An eigenvalue decomposition of the correlation matrix identified a large statistical PC1 (termed “*Global kinetics of gait pattern*”), which accounted for 36.7% of total variance. Principal component loadings also revealed a PC2 (12.6% of total variance), related to the “*Global gait regularity*.” Subsequent ANOVAs showed that only PC1 discriminated the fall status during the first 6 months, while PC2 discriminated the first fall onset between 6 and 12 months. After one year, any PC was associated with falls. These results were bolstered by the ROC analyses, showing good predictive models of the first fall during the first six months or from 6 to 12 months. Overall, these findings suggest that the performance of a standardized walking test at least once a year is essential for fall prevention.

## Introduction

In view of the high prevalence of individuals in elderly populations with a risk of falling and the potentially dramatic consequences of fall-related injuries (e.g., fractures and psychological trauma) leading to self-imposed restriction in daily activities and, consequently, loss of independence (Arfken et al., 1994; Tinetti et al., 1994a; Tinetti and Williams, 1997; Scheffer et al., 2008), avoidance or delay of the first fall onset is a major public health concern. As reported by the WHO (2007), falls are the second leading cause of accidental or unintentional-injury deaths worldwide with more than 420,000 individuals dying from falls globally, of which over 80% are in low- and middle-income countries. In addition, 37.3 million falls each year require medical attention, with about 40% of all serious fall-related injuries among the elderly resulting in hospital admission. After hospitalization, 30–40% of these patients are transferred to a nursing home. Altogether, 30% of people *over the age of 65 years* that live in the community fall at least once per year and this proportion increases greatly with age. To tackle the major public health challenges of preventing falls in older adults as soon as possible, we argue that it is crucial to identify specific markers that are significantly associated with an increased risk of the first fall onset. Identifying these markers would help the medical professional to prescribe an intervention early enough to effectively prevent a fall.

Up to now, considerable literature on the identification of risk factors for falls in the elderly and on the prediction of recurrent falls has been published (Tinetti et al., 1988; Nevitt et al., 1989; Wickham et al., 1989; O'Loughlin et al., 1993; Mahoney et al., 1994; Luukinen et al., 1995; Thapa et al., 1995; Tinetti and Williams, 1997; Lord et al., 2000; Rubenstein and Josephson, 2002; Stel et al., 2003). According to significant meta-analyses (Gillespie et al., 2012; Bloch et al., 2013), among the most important intrinsic predictors of falls are taking medications [Odds Ratio = 4.24 (3.06–5.88) 95% Confidence interval), abnormal balance test [*OR* = 2.26 (1.79–2.85)], low body mass index [*OR* = 2.05 (1.70–2.48)], fracture history [*OR* = 1.89 (1.53–2.34)], vision impairment [*OR* = 1.49 (1.39–1.59)], cardiac rhythm disorder [*OR* = 1.42 (1.14–1.75)], impaired cognition [*OR* = 1.96 (1.80–2.14)], or limited activity [*OR* = 1.32 (1.01–1.72)].

More specifically, it is well documented that a large proportion of falls in the elderly occur during walking (Wild et al., 1981; Campbell et al., 1989; Robinovitch et al., 2013). Thus, gait disturbances (e.g., decreased speed, changes in stride time variability, or medio-lateral symmetry) have been associated with falls (Rubenstein et al., 1988; Tinetti et al., 1988; Maki, 1997; Hausdorff et al., 2001; Auvinet et al., 2003; Vassallo et al., 2003; Brach et al., 2005; Verghese et al., 2009; Toebes et al., 2012; Weiss et al., 2013). For example, Mirelman et al. (2012) conducted a five-year prospective study that showed that gait disturbances, and especially dual-task gait variability, were associated with future falls, while controlling for age, gender and history of falls the year prior to the participants' initial screening. However, as far as we know, no prospective cohort study has replicated or confirmed these findings in home-dwelling people aged 65–75 *that had never fallen*. Thus our approach owes its originality both to this particular cohort (i.e., primary prevention policy) and to the compression of dimension of collected gait variables as essential information to be related to risk of the first fall in older population; In this particular context (i.e., the “unknown” cohort), we hypothesized that the average walking speed and stride regularity should be the most relevant variables to discriminate non-fallers from fallers. With an economic, easy to use, and non-invasive accelerometric device, this 2-year prospective study aimed to examine the correlations between specific gait patterns and falls. In addition, we also sought to characterize the duration between the fall-risk screening test and a potential fall using an original multi-step statistical analysis.

## Materials and methods

### Participants

A total of 259 older adults (mean age 69.6 ± 2.7 years; 61.5% women) who never had fall experience, were recruited for the present cohort, which is a prospective observational multicenter study designed to identify the risk factors for the first fall in elderly community-dwellers. This study was approved by the Local Ethical Committee of the Region of Pays de la Loire (France) (reference: n° 2004/05, “*Facteurs prédictifs du risque de première chute chez les personnes âgées* (predictive factors of risk of the first fall in elderly people); CCPPRP n°1, favorable opinion the 20th July 2004) and was conducted in accordance with the Declaration of Helsinki (last modified in 2004).

Each participant was screened by medical staff for their medical history, personal information (age, gender), physical and clinical baseline characteristics according to the aforementioned predictors of falls: taking medications, abnormal balance test (one leg standing test), body mass index, fracture history (lower limb surgery during the five past years), vision impairment (global visual acuity score), cardiac rhythm disorder (normality of electrocardiogram), impaired cognition (Mini-Mental State Examination “MMSE” and Frontal Assessment Battery “FAB” tests) and limited activity (Daily physical activity).

Eligibility criteria were age between 66 and 75 years, living at home, never fallen, and an ability to walk without assistance (for at least 30 s). Previous falls (or not) were evaluated during the first meeting with the patient, by asking them if they have already fallen. To clarify the definition of fall, the geriatric medicine doctors explained the WHO definition, with case examples. The question of fall event was discussed again with the participant during the presentation of the study and during the first baseline visit (inclusion visit). For the present analysis, exclusion criteria were refusal to give consent or lack capacity to give consent or if the participant was hospitalized at the time of screening. Participants were included after having given their written informed consent for research.

Basic gait mobility was assessed using an accelerometric device (see the *Gait assessment* section below) in order to characterize the overall locomotor behavior of each participant. After this baseline assessment, all the participants received standardized phone calls from the research medical staff each month during the first year, and every three months during the second year in order to obtain information regarding any falls or related incidents. The telephone calls were performed by trained interviewers, and were similar to the procedure used in the literature (e.g., Stalenhoef et al., 2002). By using open-ended questions, the date, circumstances, causes and consequences of falls, and changes in living conditions and life events were collected. If necessary, the interviewers reminded the WHO definition to the participants, with case examples. A fall was defined as “unintentionally on the ground or lower level, not as a result of a major intrinsic event (such as a stroke) or overwhelming hazard” (Tinetti et al., 1988; WHO, 2007).

### Categorization of fallers groups according to the date of the first fall onset

At the end of the follow-up period, a committee of geriatric medicine doctors analyzed the circumstances of each fall recorded during the prospective follow-up in order to verify and, if appropriate, validate that the fall occurred during usual living conditions and was factually consistent with the definition-related criteria of a fall (WHO, 2007). For example, a fall occurring while practicing a high-risk sport or because of ice on the sidewalk was not considered as a falling event. About 5% of collected falls were rejected by this committee of experts. Three participants were then excluded from this study before the analysis. During the 24-month follow-up period, 72 subjects (27.40%) reported falling one or more times. Among the reported first falls, 20 participants (7.72%) fell during the first six months, 26 (10.04%) between the sixth and twelfth months and 26 (10.04%) during the second year. 69 of the 72 subjects that fell reported falling only once and 3 fell multiple times during the follow-up period. Note also that the committee kept blind for the gait assessment results as the geriatric M.D. met. In addition, none of them has been involved in the statistical process.

### General baseline characteristics

According to the main factors identified involved in the risk of falling in older people (Gillespie et al., 2012; Bloch et al., 2013), the relationship between falls and these potential confounding factors was examined by means of multinomial logistic regression analysis. By performing this statistical analysis for each variable separately, we specified the association between (the risk of) the first fall and these clinical factors, with the non-fallers group used as the reference level. The baseline clinical characteristics were gender, taking medications, daily physical activity (above 30 min per day), one leg standing test (above 5 s), lower limb surgery during the five previous years, and abnormal electrocardiogram *as binary variables*, and age, body mass index, global visual acuity score, and MMSE and FAB tests as parametric variables.

### Accelerometric gait analysis device

**Figure 1:**
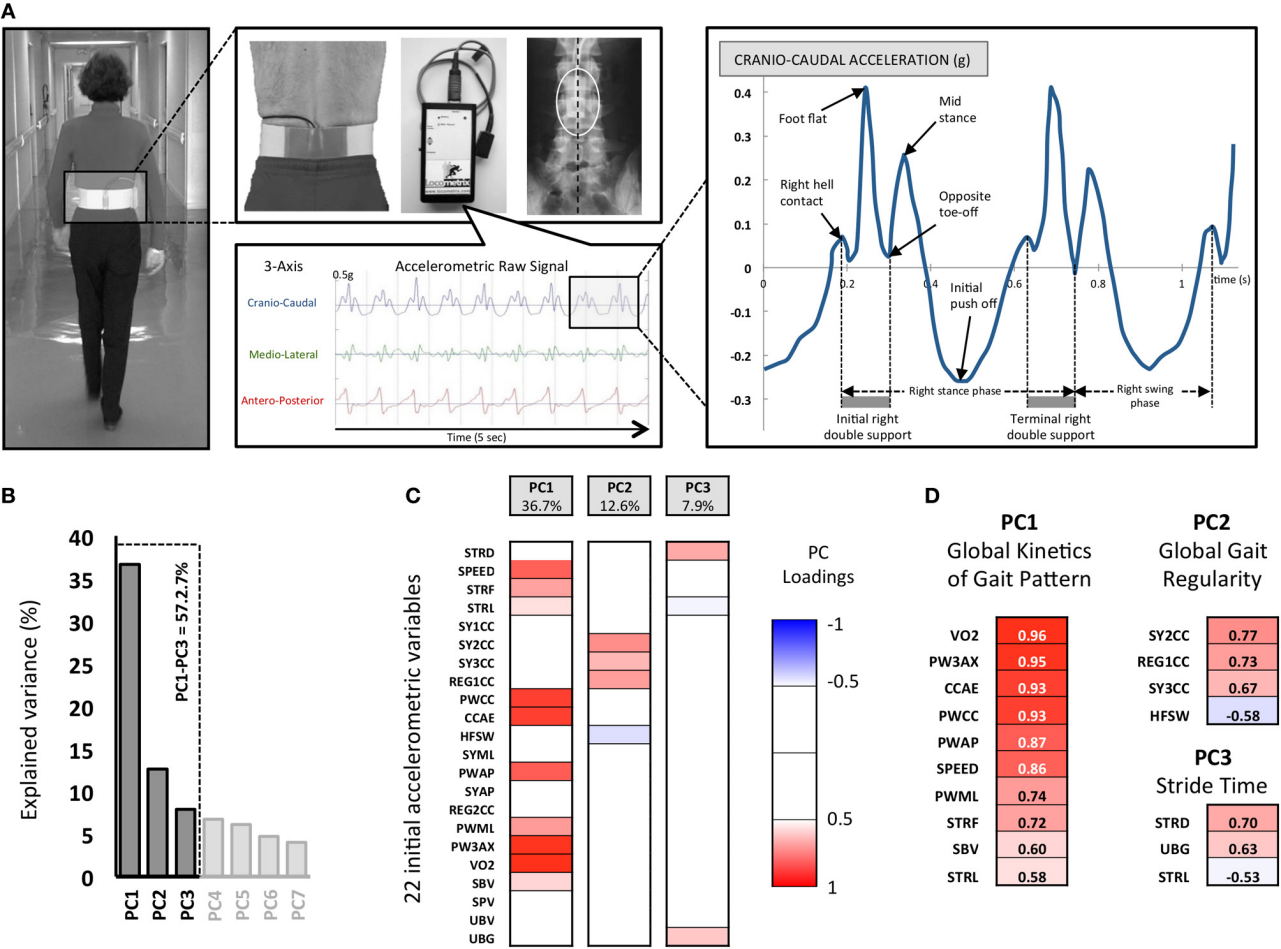
**The experimental Locometrix® gait analysis system for the walking test and first methodological steps of the Principal Components Analysis (PCA)**. **(A)** The accelerometric sensor is applied in the middle of the lower back using an elastic beltfastened around the subject's waist. The sensors are connected to a data logger, which is attached onto the front part of the belt. The participants were requested to walk at their own comfortable speed along a 30 m straight corridor. The sensor provides the cranio-caudal, medio-lateral and antero-posterior raw acceleration signals. Then the software allowed to select walk sample of 20 s to calculated 22 variables related to kinetics, regularity, power and expended energy of locomotor behavior. **(B)** 22792 pieces of data corresponding to 22 variables, extracted from 1036 walking trials, were used for the PCA. The three retained principal components (PC) are shown with their associated eigenvalues, for 57.2% of the total variance. **(C)** Each initial variable, as correlated with a PC with |r| > 0.5 (*p* < 0.05), was considered “significant” and used for interpretation. The color code corresponds to these component loadings. **(D)** The resulting analysis identified three groups of variables that can be related to (i) the *Global kinetics of gait pattern* (PC1), (ii) *Global gait regularity* (PC2) and (iii) the *Stride time* (PC3).

The gait analysis system used in this study included a 3-D-acceleration sensor, a data logger and a computer program for processing the acceleration signals and calculating the gait parameters (Locometrix®, Figure 1). The sensor weighs 20 g and is composed of three accelerometers placed perpendicularly to each other and housed in a moulded box (40 × 18 × 18 mm). The sensor is incorporated into an elastic belt, which was fastened around the subject's waist, so that the sensor was placed over the L3–L4 inter-vertebral space (Figure 1). The first accelerometer was aligned with the cranio-caudal axis of the body, the second one with the antero-posterior axis and the third one with the medio-lateral axis. Signals were recorded by a data logger at a sampling frequency of 100 Hz and an anti-aliasing filter with a cut-off frequency of 50 Hz was applied. This data logger weighed 140 g and was housed in a box (65 × 22 × 12 mm) (see Auvinet et al., 1999, 2002, for further details).

### Gait assessment

Four tests were carried out on each subject walking at his/her own comfortable speed down and back along a 30 m straight hospital corridor. No prompting signals were used. The 30 m distance was long enough to ensure a constant speed over 25 s in order to select a steady state walking pattern of 20 s for analysis. All subjects wore their usual walking shoes avoiding high heels or hard-soled shoes. The walking speed was measured with an electronic stopwatch synchronized with the gait data logger. Although subjects were asked to walk in a straight line with their arms free, the trajectory was not imposed and the corridor width was limited so the conditions of walking allowed a large degree of freedom for walk disorder expression with the environmental conditions carefully standardized. Overall, considering the 259 subjects, 1036 tests of 30 m distance have been recorded or approximately 30 kilometers and 60000 strides.

### Gait variables

The software program Locometrix® automatically calculated kinematic and kinetic gait variables after selecting a steady state walk sample of 20.48 s. This sample included exactly 1024 points of acceleration measurements on each axis, which provided an optimal calculation for Fast Fourier Transformation (FFT) and other algorithms. This period corresponded to 19–21 walk cycles-about 28 m for healthy adult subjects. All the biomechanical variables were calculated from the walk sample 3D-accelerations signals for each person. These variables were derived from calculating of several algorithms as indicated in Table 1: time measure, vectorial calculations, FFT, autocorrelation, wavelet analysis and statistical regressions. Further explanations on the calculation of the variables can be obtained in previous validation papers of the accelerometric gait analysis device (Auvinet et al., 1999, 2002). This gait analysis system has been extensively used for clinical trials both in humans and animals (Paquet et al., 2003; Auvinet et al., 2011; Barthélémy et al., 2011). The 22 collected variables are presented in detail in Table 1.

**Table 1:** **Details of 22 gait variables collected from the accelerometric gait analysis device**.

**Short title**	**Full title**	**Units**	**Methods**	**Brief definition**
SPEED	Speed	m/s	Regression model	Average linear speed of forward displacement
STRF	Stride frequency	Hz	Fast Fourier Transform	Number of walk cycles per unit of time
STRD	Stride duration	s	Time measure	Average duration between two successive ground contact of the same foot
STRL	Stride length	m	Speed/SF	Average length between two successive ground contact of the same foot
PWCC	Power in CC axis	W/kg	Fast Fourier Transform	Power extracted from the FFT spectrum in CC axis
PWAP	Power in AP axis	W/kg	Fast Fourier Transform	Power extracted from the FFT spectrum in AP axis
PWML	Power in ML axis	W/kg	Fast Fourier Transform	Power extracted from the FFT spectrum in ML axis
PW3AX	Total mechanical power on the 3 axes	W/kg	Combination	Sum of the 3 powers extracted from the FFT spectrum in CC, AP, LM axes
VO2	Oxygen consumption estimate	ml/min/kg	Regression model	Estimation of oxygen consumption based on high correlations between VO2 and power and SF variables
SBV	Support and breaking vector	g	Vector calculation	Averaged vector of the first part of support phase (deceleration or breaking phase)
SPV	Support and propulsion vector	g	Vector calculation	Averaged vector of the second part of the support phase (propulsion)
UBG	Unloading and breaking vector	g	Vector calculation	Averaged vector during unloading and breaking phase
UBV	Unloading and propulsion vector	g	Vector calculation	Averaged vector during unloading and propulsion phase
SY1CC	Symmetry index 1 CC axis	Without	Autocorrelation	Comparison of the left and right acceleration patterns on CC axis (both acceleration amplitude and time on all the strides)
SY2CC	Symmetry index 2 CC axis	Without	Wavelet analysis+autocorrelation	Comparison of the left and right acceleration patterns on CC axis (both signal energy and time over all the sample)
SY3CC	Symmetry index 3 CC axis	Without	Wavelet analysis+autocorrelation	Comparison of the left and right acceleration patterns on CC axis (both signal energy and time on all the strides)
SYML	Symmetry index 4 ML axis	Without	Wavelet analysis+autocorrelation	Comparison of the left and right acceleration patterns on ML axis (both signal energy and time on all the strides)
SYAP	Symmetry index 2 AP axis	Without	Wavelet analysis+autocorrelation	Comparison of the left and right acceleration patterns on AP axis (both signal energy and time on all the strides)
REG1CC	Regularity index 1 CC axis	Without	Autocorrelation	Variability analysis of the pattern in successive strides of the sample by analysis of the acceleration patterns in CC axis
REG2CC	Regularity index 2 CC axis	Without	Wavelet analysis+autocorrelation	Variability analysis of the pattern in successive strides of the sample by analysis of the signal energy patterns in AP axis
CCAE	CC acceleration energy	J/	Wavelet analysis	Total Energy of the wavelet spectrum on CC acceleration signal
HFSW	High frequency shock wave	%	Wavelet analysis	Percentage of the total energy due to high frequency >4Hz due to foot impacts and transient

### Data and statistical analysis of gait

We tried to characterize the overall gait behavior for the different (non) fallers groups by performing a statistical analysis consisting of a seven-step procedure. Specifically, we replicated the statistical process mainly based on a principal components analysis (PCA) as described in recent studies (Courtine et al., 2009; Musienko et al., 2011; van den Brand et al., 2012).

#### Step 1

A total of 22 gait variables providing detailed quantification of the overall locomotor behavior from the 3-axis accelerometric device that were collected (Figure 1).

#### Step 2

A PCA was applied on all computed variables. This PCA allowed the extraction of the most relevant information from the initial data by generating new independent variables called Principal Components (PC). Each PC linearly combines the original variables to maximize the amount of explained variance for each successive PC (Figure 1).

#### Step 3

We then computed correlations (PC loadings) between each measured parameter and each selected PC. In order to understand what each PC reflected, we focused on the variables that showed the highest PC loading (*r* > 0.5, *p* < 0.05). PC1 accounted for the largest part of the variance (36.7%), PC2 for 12.6% of the total variance and PC3 for 7.9% of the total variance) (Figure 1). Note that these three PCs are sufficient to summarize the entire collected data because there is a very strong correlation (*r* = 0.92, *p* < 0.001) between the eigenvector constructed with these first three PCs and the eigenvector constructed from all the PCs (i.e., 100% of the variance).

#### Step 4

**Figure 2:**
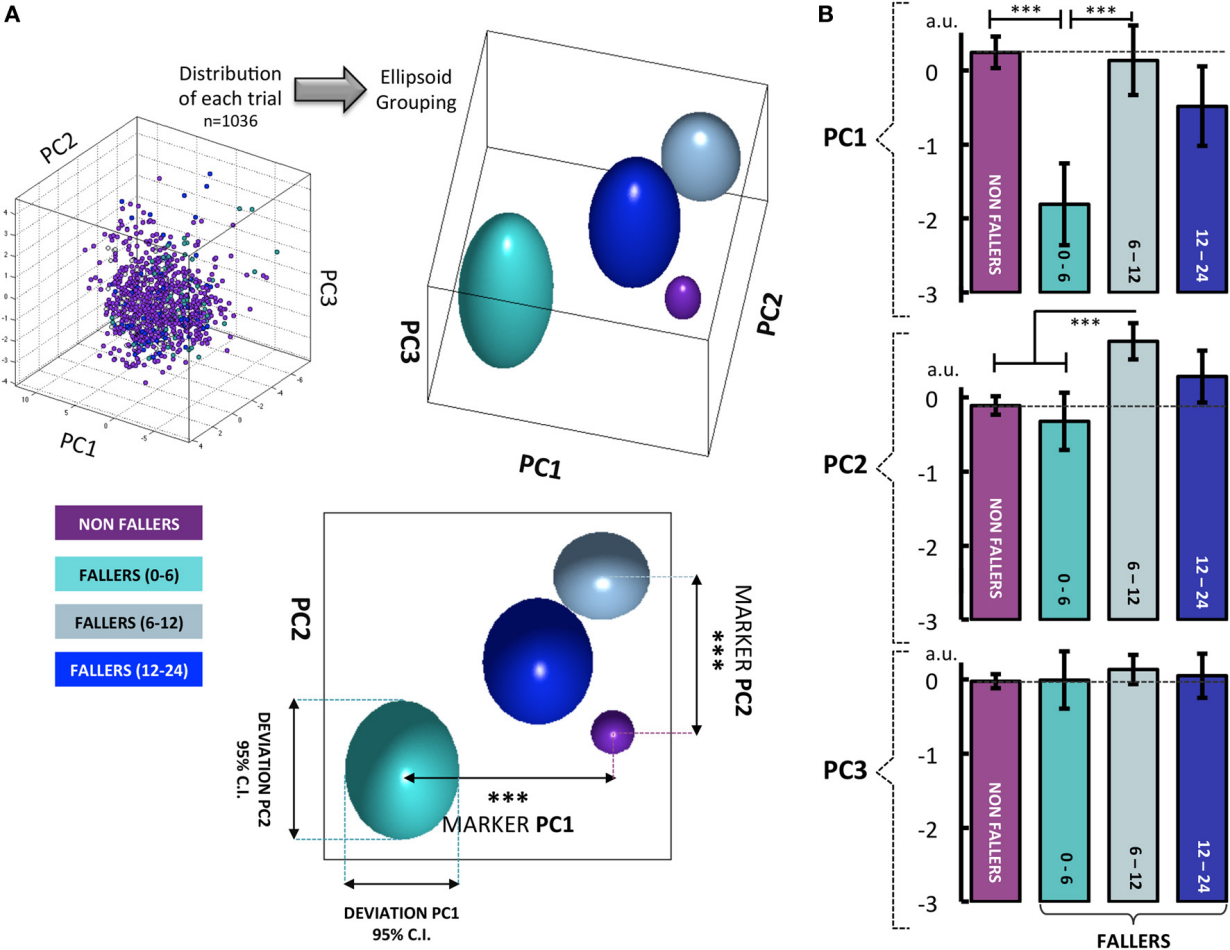
**(A)** When results from all walking tests are visualized in a 3-D space defined by the newly constructed variables PC1–3 (57.2% of explained variance), clear differences can be seen between the four groups: non-fallers, fallers from 0 to 6 months, fallers from 6 to 12 months and fallers from 12 to 24 months. For the PC1, the participants who fell from 0 to 6 months show a clear difference in behavior compared to the three other groups. Similarly, when considering the PC2, the fallers from 6 to 12 months can be significantly differentiated from all the other groups. With PC3, no differentiation between groups was found. Note that the group of fallers from 12 to 24 months displays a behavior very similar to the non-fallers group. **(B)** These visual findings are confirmed by one-way analysis of variance, with the 4 groups used as a differential factor between subjects. The HSD Tukey tests were used as *post-hoc* tests following significant effects. The histograms represent the mean score of eigenvector for each group with ±95% confidence intervals. *Note*. ^*^p < 0.05, ^**^p < 0.01 ^***^p < 0.001.

The overall locomotor behavior of each group was then displayed in a 3-D space defined by the newly constructed variables, PC1–3 (57.2% of explained variance) (Figure 2). Note that clear differences emerged visually across the groups. The displayed ellipsoid volumes for each group were located from the mean value of the three PC eigenvectors for each group, and their diameters corresponded to the 95% confidence interval (95% CI).

#### Step 5

The expected differences between groups were represented by histograms (Figure 2), which correspond to the mean position ±95% CI, according to the three PC eigenvectors. One-Way analysis of variance (ANOVA) was used to examine the between-groups effects with each eigenvector as dependent variable. This fifth step allowed for the identification of the delay-related predictive marker of the first fall onset.

#### Step 6

**Figure 3:**
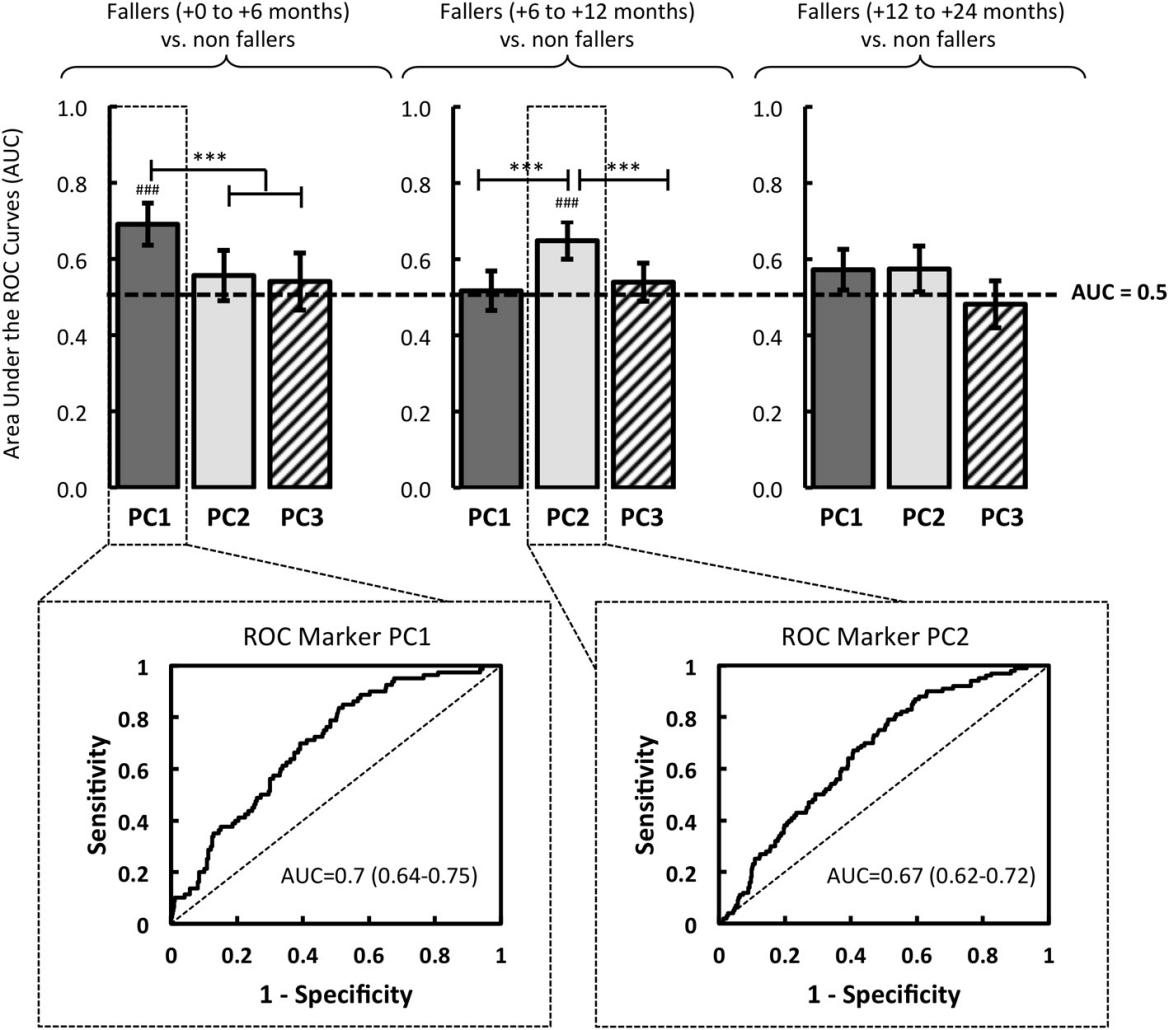
**Based on logistic regressions performed with the eigenvectors of PC1, PC2 and PC3, the quality of the models was evaluated by the area under the Receiver Operator Characteristic (ROC) Curve (AUC)**. The resulting models were compared in relation to three groups of fallers: fallers from 0 to 6 months, fallers from 6 to 12 months, and fallers from 12 to 24 months. The significant AUC (i.e., different from a random law) are displayed in the figure where ^#^*p* < 0.05, ^##^*p* < 0.01, or ^###^*p* < 0001. *Note*. Significant difference between models is reported: ^*^p < 0.05, ^**^p < 0.01 ^***^p < 0.001.

In order to specify the relationships between the occurrence of the first fall event during the follow-up and extracted PCs (step 5), we assessed the Area Under the Curve (AUC) of the Receiving Operating Characteristics (ROC), plotted from the sensitivity and sensibility of the PCs eigenvectors, for the three fallers groups in comparison *to the non-fallers group*: fallers (+0 to +6 months) vs. non-fallers, fallers (+6 to +12 months) vs. non-fallers, fallers (+12 to +24 months) vs. non-fallers (Figure 3).

#### Step 7

Lastly, Odds ratios (OR) were quantified by performing multiple univariate logistic regressions after a dichotomization process, necessary to transform the continuous eigenvectors, by the computation of the *Youden Index* (Youden, 1950; Shapiro, 1999; Greiner et al., 2000). The latter consisted of the determination of the cut-off value (J), from a maximization process of (Sensibility + Specificity) − 1; {*J* = maxc [Se(c) + Sp(c) −1]}. On this basis, survival curves analyses using Kaplan-Meier testing were performed to assess if the identified markers were significantly associated to fall as a function of its occurrence delay.

## Results

### Baseline clinical characteristics

The mean and standard deviations, or frequencies and percentages, as appropriate, of the baseline characteristics of the entire sample and the four groups [non-fallers, fallers (+0 to +6 months), fallers (+6 to +12 months) and fallers (+12 to +24 months)] are presented in Table 2. To identify potential differences between non-fallers and fallers, One-Way ANOVAs (or equivalent non-parametric Kruskal-Wallis test as appropriate) were performed for all clinical variables. Note that no main effect of group was found, whatever the tested clinical variable. In addition, the overall statistical results are presented for each of these confounding variables in Table 3, with the regression coefficient β ± the standard deviation and *p*-value. It is worthy to note that only a significant association between falls and age was found for the fallers group (+0 to +6 months), with the non-fallers used as the reference level (β = 0.23 ± 0.09; *p* = 0.01). There were no other significant relations, no matter what the confounding factor included in the multinomial logistic regression analysis.

**Table 2:** **Baseline clinical characteristics (mean ± standard deviation *SD*, or percentages) of the entire sample and the four groups: non-fallers, fallers from 0 to 6 months, fallers from 6 to 12 months and fallers from 12 to 24 months**.

	**Full sample (***n*** = **259**)**	**Non fallers (***n*** = **187**)**	**Fallers (+0 to +6 months) (***n*** = **20**)**	**Fallers (+6 to +12 months) (***n*** = **26**)**	**Fallers (+12 to +24 months) (***n*** = **26**)**
Gender (woman, %)	152(58.7)	115(61.5)	10(50)	12(46.2)	15(57.7)
Age (years ± *SD*)	69.5 ± 2.6	69.4 ± 2.5	71.1 ± 2.7	69.3 ± 2.8	69.2 ± 2.5
Body mass index (kg.m^−2^ ± *SD*)	26.1 ± 3.6	26 ± 3.6	26.6 ± 3.8	26.2 ± 3.8	26.5 ± 3.2
Taking medications (%)	209(81)	145(78)	17(85)	24(92.3)	24(92.3)
Daily physical activity > 30 min (%)	201(77.6)	148(79)	15(75)	20(76.9)	18(69.2)
Global visual acuity score (a.u. ± *SD*)	1.7 ± 5.9	1.7 ± 6.0	0.6 ± 0.2	2.9 ± 9.5	0.9 ± 0.8
MMSE (score/30 ± SD)	27.2 ± 2.5	27.1 ± 2.4	27.1 ± 3.0	27.4 ± 2.5	27.1 ± 2.4
FAB (Score/18 ± *SD*)	13.9 ± 2.7	13.8 ± 2.6	13.4 ± 2.6	14.2 ± 3.3	13.9 ± 3.1
One leg standing > 5 s (%)	225(87.2)	164(87.5)	16(80)	22(84.6)	23(88.4)
No lower limb surgery (%)	220(85.3)	160(85.4)	15(75)	22(84.6)	23(88.4)
Not abnormal electrocardiogram (%)	202(78.3)	151(80.6)	13(65)	18(69.2)	20(76.9)

**Table 3:** **Regression coefficient β ± standard deviation and *p*-value for all baseline characteristics, obtained by multinomial logistic regression analysis**.

	**Non fallers = *reference* (***n*** = **187**)**					
	**Fallers (+0 to +6 months) (***n*** = **20**)**		**Fallers (+6 to +12 months) (***n*** = **26**)**		**Fallers (+12 to +24 months) (***n*** = **26**)**	
	**β ± *SD***	***p***	**β ± *SD***	***p***	**β ± *SD***	***p***
Gender	0.47 ± 0.47	0.320	0.62 ± 0.42	0.139	0.16 ± 0.43	0.707
Age	0.23 ± 0.09	**0.010**	−0.02 ± 0.08	0.805	−0.03 ± 0.08	0.750
Body mass index	0.05 ± 0.06	0.472	0.02 ± 0.06	0.734	0.04 ± 0.06	0.495
Taking medications	0.44 ± 0.65	0.504	1.19 ± 0.76	0.118	0.74 ± 0.64	0.249
Daily physical activity (>30 min)	0.27 ± 0.55	0.626	0.16 ± 0.50	0.746	0.55 ± 0.46	0.231
Global visual acuity	−0.05 ± 0.09	0.558	−0.09 ± 0.08	0.275	0.03 ± 0.08	0.743
MMSE	0.01 ± 0.17	0.959	0.09 ± 0.18	0.625	0.07 ± 0.17	0.693
FAB	−0.055 ± 0.08	0.496	0.057 ± 0.083	0.490	0.006 ± 0.078	0.938
One leg standing (>5sec)	0.68 ± 0.68	0.322	0.71 ± 0.61	0.245	−0.07 ± 0.78	0.924
No lower limb surgery	0.89 ± 0.56	0.117	0.28 ± 0.59	0.635	−0.05 ± 0.65	0.936
Not abnormal electrocardiogram	0.78 ± 0.53	0.141	0.81 ± 0.47	0.084	0.42 ± 0.51	0.408

*Note. Significant results are indicated in bold type (i.e., p < 0.05)*.

#### Steps 1–4

PC1 is composed of the following variables (*r* > 0.5, *p* < 0.05), which represent 80.6% of the PC1-variance and 36.7% of the total variance: *VO2, PW3AX, CCAE, PWCC, PWAP, SPEED, PWML, STRF, SBV, STRL* (see Figure 1 and Table 1 for the details). We denominated this PC1 the “*Global kinetics of gait pattern*,” including the mechanical power and temporo-spatial variables of walking gait. Principal component loadings also revealed a PC2 called “*Global gait regularity*,” which is composed of following variables (*r* > 0.5, *p* < 0.05), representing 54.3% of the PC2-variance and 12.6% of the total variance: *SY2CC, REG1CC, SY3CC, HFSW* (see Figure 1 and Table 1 for details). This component included three variables related to gait symmetry and regularity of walking strides (low variability). Finally, PC3 is constituted of variables (*r* > 0.5, *p* < 0.05) which represent 49.7% of the PC3-variance and 7.9 % of the total variance: *STRD, UBG, STRL* (see Figure 1 and Table 1 for details). We labeled PC3 “*Stride time*,” including both stride duration and sum of acceleration vectors averaged by a part of stride time duration.

#### Step 5

The decomposition of the PC eigenvectors according to the non-faller groups showed a significant effect of group for PC1 [*F*_(3, 1032)_ = 13.58, *p* < 0.001] and PC2 [*F*_(3, 1032)_ = 10.06, *p* < 0.001]. No main effect of the group was shown with PC3 [*F*_(3, 1035)_ = 0.48, *p* = 0.7]. The following HSD-Tukey *post-hoc* tests showed that the fallers (+0 to +6 months) are greatly different from the non-fallers (*p* < 0.001) and the fallers (+6 to +12 months) (*p* < 0.001) on PC1. When considering PC2 “*Global gait regularity*,” the *post-hoc* comparisons revealed that the fallers (+6 to +12 months) differed from the non-fallers (*p* < 0.001) and the fallers (+0 to +6 months) (*p* < 0.001) (Figure 2).

#### Step 6

The ROC analysis revealed that PC1 had a significant predictive power of the first fall onset during the first six months after the initial screening: AUC = 0.7 (0.64–0.75, 95% CI) (*p* < 0.001). Over the course of these first six months, no association between the PC1 and falls was found (AUC < 0.5, *p* > 0.05) (Figure 3).

In the same vein, the logistic regression analysis revealed a relevant interest of PC2, significantly associated with an increased risk of first fall onset only when it occurred between the sixth and the twelfth months: AUC = 0.67 (0.62-0.72, 95% CI) (*p* < 0.001) (Figure 3). Note that no predictive power of PC3 was determined by the ROC analysis (AUC < 0.5, *p* > 0.05), whatever the date of the first fall onset.

#### Step 7

**Figure 4:**
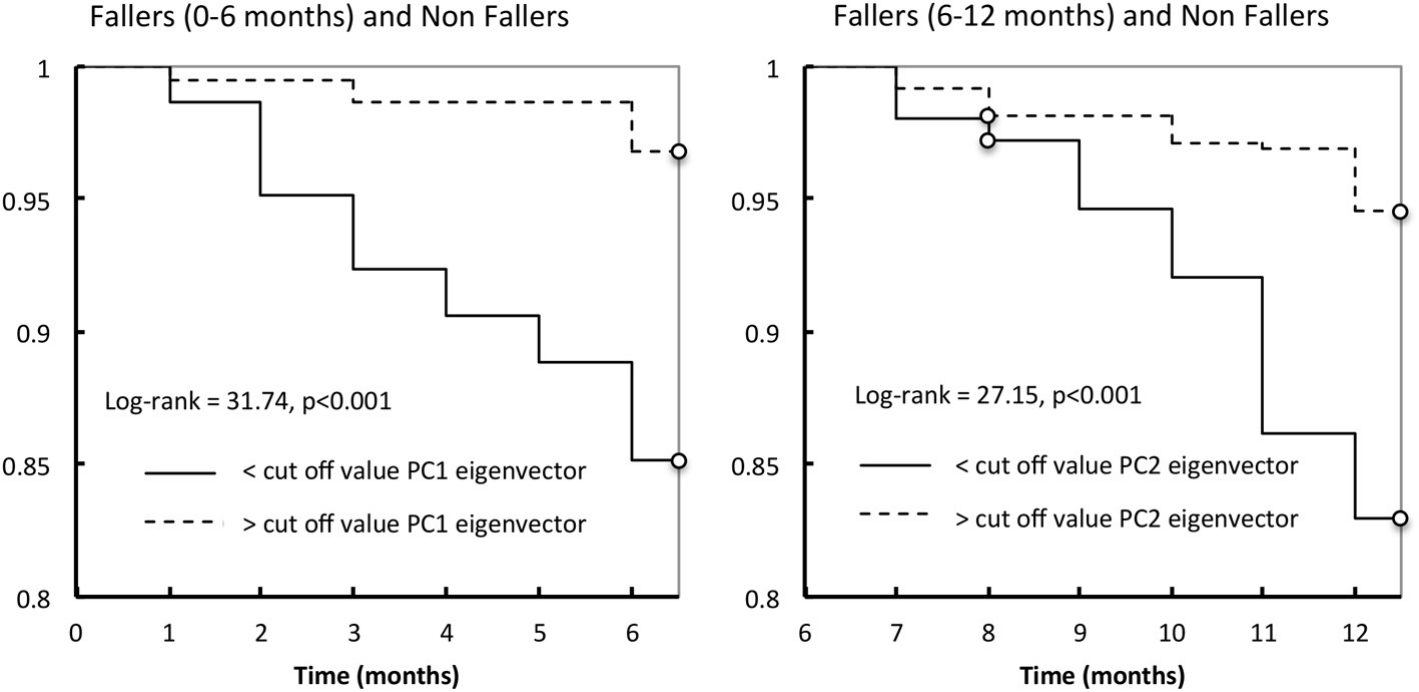
**Kaplan-Meier estimates of the probability of a first fall occurrence during the first year follow-up according to the cut-off value of PC1 eigenvector (left) or PC2 eigenvector (right)**.

The logistic regression parameters showed ORs of 3.89 (2.2–6.7, 95% CI) (*p* < 0.001) and of 3.6 (2.16–5.89, 95% CI) (*p* < 0.001), for PC1 and PC2, respectively. Lastly, considering only the non-fallers and fallers (+0 to +6 months), Kaplan-Meier's distributions of falls differed significantly between those with values lower than cut-off value PC1 eigenvector and those with values higher than the cut-off value PC1 (log-rank test = 31.74, *p* < 0.001; Figure 4). For the non-fallers and fallers (+6 to +12 months), similar difference of Kaplan-Meier's distributions of falls was found between those with values lower than cut-off value PC2 eigenvector and those with values higher than the cut-off value PC2 (log-rank test = 27.15, *p* < 0.001; Figure 4).

## Discussion

Using a very simple and economical gait analysis system that allowed ecological measurements, we proposed a metrological and statistical analysis of gait patterns in home-dwelling people aged 66–75, with follow-up telephone calls each month during the first year, and every three months during the second year. The current findings provide for the first time the possibility of identifying relevant indicators of imminent fall occurrence, that is the “*Global kinetics of gait pattern*” (PC1) and the “*Global gait regularity*” (PC2). In addition to the fact that the identification of these markers is relatively easy to envisage in clinical settings (i.e., safe test, short duration, and low cost), and might of special interest to accurately estimate the available time before the occurrence of the first fall. Furthermore, this new key information might be useful for recommending a specific fall-prevention program. From a clinical viewpoint, to perform a walking test at least once a year might be essential for fall prevention.

It is important to bear in mind that the current cohort is very specific and original (i.e., home-dwelling people that had never fallen), and by definition has no previous evidence. In this respect, it is not necessarily surprising that no difference between the four groups was found when considering all the screened clinical variables (see Table 3). It might be suggested that the present population was “full-matched” at the time of inclusion, with the same well-documented multifactorial risks for falling (Gillespie et al., 2012; Bloch et al., 2013). Thus our current findings on the specific gait markers reinforce the main idea to accurately assess gait behavior for this healthy home-dwelling population. But this point needs to be confirmed and to be precisely tested in a prospective independent cohort. Nevertheless the current findings observed in an original cohort confirm that the gait analysis is probably one of best tools to predict the first fall onset, and most importantly, its occurrence time frame.

### Specific gait markers and risk of first fall

Numerous studies have already demonstrated the relationship between gait disorders and risk of falling in the elderly, by supporting the idea that a decrease in walking speed in usual conditions and an increase of stride-time variability are strong predictors of falls (Dargent-Molina et al., 1996; Brach et al., 2005; Verghese et al., 2009; Studenski et al., 2011). However, as far as we know, all the conclusions drawn in the current retro- or prospective studies cannot be implemented in a population of healthy elderly people that have never fallen. Indeed, studies with prospective follow-up for falls do not warrant that the participants included in the cohorts had not already fallen before the year preceding the initial screening.

In this original context, the alterations in *global kinetics of gait pattern* (i.e., the PC1) can be used as a new locomotor marker, which is significantly associated with an increased risk of imminent fall occurrence. Based on a data reduction of high-dimensional gait data to a low-dimensional set of essential features, our analysis actually showed that PC1 discriminated the fall status during the first 6 months (see Figures 2, 3). Moreover, these results are bolstered by the ROC analyses: the logistic model showed that PC1 was significantly related to a higher risk of first fall during the first six months (AUC = 0.7, *p* < 0.001, OR = 3.89, *p* < 0.001). It is also worth noting that this first marker is modeled by a few variables (VO2: *r* = 0.957, *p* < 0.001, PW3AX: *r* = 0.952, *p* < 0.001 and CCAE: *r* = 0.927, *p* < 0.001), which are closely linked to symptoms of hypokinesia (see Table 1). Indeed, it is well established in the literature that aging can lead to impairments in the central and/or peripheral nervous system, which are consequently reflected in executive functions, or by a decrease in physical, functional and locomotor performance (e.g., Lundin-Olsson et al., 1997).

In this light, the slowing down of walking speed—a variable that composes the PC1—might be partially explained by a decrease in volume / thickness of white and gray matter at the cortical and subcortical levels (i.e., corticospinal tract, cortical atrophy in the frontal, parietal, hippocampal and motor-cortex), which are in charge of the programming and the execution of locomotor commands (Annweiler and Montero-Odasso, 2012; de Laat et al., 2012; Dumurgier et al., 2012; Rosano et al., 2012). Moreover, considering pathways downstream of the CNS, physiological aging also causes alterations in nerve conduction velocities (Borg, 1981; Wang et al., 1999; Scaglioni et al., 2002), muscle synergies/coordination (Woollacott et al., 1986; Olafsdottir et al., 2007), contractility of acto-myosin bridges (Frontera and Bigard, 2002; D'Antona et al., 2003), and transmission of force to the skeletal system (Narici and Maganaris, 2006; Onambele et al., 2006; Carroll et al., 2008). All these changes might cause impairment in motor skills, postural control and gait speed (Baloh et al., 1998; Brach and VanSwearingen, 2002; Amiridis et al., 2003; Capodaglio et al., 2005; Buatois et al., 2006; Jung et al., 2006; Paterson and Warburton, 2010). Taken together, these aforementioned studies might explain the observed hypokinetic behavior in participants that have fallen during the first 6 months, as an evidence of lack of flexibility (Hausdorff et al., 1996; Jordan et al., 2007).

The second major result highlighted in this study is the relevance of the “*Global gait regularity*” marker strongly related to the occurrence of a possible first fall 6–12 months after the initial screening test (AUC = 0.67, *p* < 0.001, *OR* = 3.6, *p* < 0.001). Given the three variables included in the PC2 (these variables are SY2CC: *r* = 0.766, *p* < 0.001, REG1CC: *r* = 0.733, *p* < 0.001 and SY3CC: *r* = 0.675, *p* < 0001), the locomotor behavior is characterized by changes in regularity and symmetry parameters.

There is clear evidence to suggest that the *global gait regularity* or gait variability (usually reported by using the coefficient of variation of stride time) is strongly associated with a risk of falling (Brach et al., 2005; Montero-Odasso et al., 2009; Lord et al., 2011; Beauchet et al., 2012; Toebes et al., 2012). From a neurophysiological point of view, some studies have clearly shown that changes in gait variability could be the result of atrophy and dysfunction in the parietal cortex (right angular gyrus) and hippocampus, which supports the idea of the decline of sensorimotor areas involved in executive functions (Camicioli et al., 1997; Marquis et al., 2002; Hausdorff, 2005; Zimmerman et al., 2009; Zwergal et al., 2012; Beauchet et al., 2013). More precisely, according to the results of Beauchet et al. (2013), the focal neurodegeneration (especially in the parietal cortex, which is strongly involved in executive functions) might have altered the spatial displacement in relation to the surrounding environment, with subsequent impairment in gait regularity. In the same vein, Zwergal et al. (2012) demonstrated a relationship between age and the cortical control of gait, evidenced by higher attentional cost for controlling locomotor activity with age (i.e., the component of voluntary and executive function processes during the control of gait). In particular, they showed that the supraspinal locomotor centers remained preserved during aging, but multisensory cortical control of locomotion changed with age. If young people adopt an automated mode of locomotion, the multisensory cortical activation in elderly persons occurs as a result of reduced reciprocal inhibitory sensory interaction. This might serve as a compensatory mechanism for peripheral sensory decline with age and confirm the more costly and irregular mode of locomotion in the elderly (see Zwergal et al., 2012, for details).

### What could be done with these significant markers?

The aforementioned morpho-functional alterations in muscular peripheral and central nervous systems as the source of gait disturbances are the result of natural physiological aging and apoptosis (Lexell, 1997; Tomlinson and Irving, 1977). Despite the inevitable component of aging, it is, however, possible to reduce or delay this age-related cognitivo-motor decline, through the preservative effects of adapted and regular physical activity. In fact, many studies have already demonstrated the positive impact of different exercise programs (e.g., voluntary muscular strength, neuromuscular electrostimulation, aerobic exercises, functional daily living exercises, combined training, dancing) on the daily activities and autonomy, quality of life, balance and/or walking (Fiatarone et al., 1994; Tinetti et al., 1994b; Dionne et al., 2003; Gauchard et al., 2003; Robinson et al., 2004; Toulotte et al., 2004; Capodaglio et al., 2005; Paillard et al., 2005; Sievänen and Kannus, 2007).

A subject exhibiting a generalized hypokinesia (i.e., indicative of imminent fall occurrence a maximum of 6 months prior to falling), could have an urgent need to be included in a prevention program. For example, the neuromuscular changes following a muscular training program are well-documented in the literature in order to improve locomotor skills (Scaglioni et al., 2002; Capodaglio et al., 2005; Paillard et al., 2005). In parallel, or perhaps a priority given this patient profile, the implementation of structured safety programs for the daily living environment is of special interest for reducing risk factors (Tinetti et al., 1994b; Cumming et al., 1999; Sievänen and Kannus, 2007).

A subject with alterations in gait regularity and symmetry (i.e., the “*gait variability*” marker linked to the occurrence of a potential first fall 6–12 months), would have enough time to benefit from specific therapeutic actions (multi-localized actions leading to a more efficient adaptation). This suggestion is in agreement with a recent study by Trombetti et al. (2011), which showed the benefits of a 6-month exercise multitasking program (performed at the tempo dictated by piano music), on the recovery of gait variability at normal levels, associated with a significant reduction of falling risk in the elderly.

## Limits and concluding remarks

Some limitations of the present study need to be considered. First, it should be noted that the number of fallers group was relatively low for each subgroup: *n* = 20 for fallers (+0 to +6 months), *n* = 26 for fallers (+6 to +12 months) and fallers (+12 to +24 months), and the size of fallers sample should be increased to reinforce the predictive power of our first risk profile model. The possibility of over-fitting results needs to be considered. It is worth reminding that each subject performed four gait tests. Thus each walking test was considered as “a subject” that can be implemented into the PCA and subsequent statistical analyses (Courtine et al., 2009; van den Brand et al., 2012). Accordingly, all the analyses have been performed by considering 748 nonfallers-related tests and 288 fallers-related tests (i.e., 1036 “walking tests”). When fallers have been splitted in subgroups, then the analyses have been performed with *n* = 80 fallers-related tests for 0–6 months, or *n* = 104 fallers-related tests for 6–12 months or >12 months. This validated methodological procedure provided the advantage to reinforce the internal validity of results. Secondly, the regular follow-up phone-calls did not allow for re-assessment of the participants, in particular with regard to the walking test, in order to check what parameters were altered after the fall. Lastly, this observational design did not allow for the control of the risk factors and events during the follow-up portion of the study. Even if there is a significant association between gait parameters and future falls (with potential clinical impact in terms of recommendations for specific fall-prevention programs), no causal link between the currently observed indicators and the first fall onset can clearly be drawn.

In any event, for the first time, the alterations in *global kinetics of gait pattern* and *gait regularity* have been identified as locomotor markers in older people that had never fallen. We suggest that these two specific gait markers might help the medical profession to prescribe an intervention early enough to effectively prevent a fall in *healthy* elderly people. Within this context of effective primary prevention, medical professionals could also recommend urgent changes in the patient's environment and recommend structured safety programs that will target suboptimal practices for environmental and personal safety.

### Conflict of interest statement

Prof. Eric Barrey and Dr. Bernard Auvinet developed and validated the gait analysis software Locometrix® for medical and sport applications. This device has been patented by INRA and is sold under exclusive license by the company Centaure Metrix. The other authors declare that the research was conducted in the absence of any commercial or financial relationships that could be construed as a potential conflict of interest.
